# Exploring Workplace Learning in Surgical Practice: How Mindset and Motivation Are Associated With Self-Regulated Learning Behaviors

**DOI:** 10.5334/pme.2144

**Published:** 2025-11-03

**Authors:** Kirsten Felicia Ann-Sophie Aimée Dabekaussen, Gepke L. Veenstra, Manja Vollmann, Kiki M. J. M. H. Lombarts, Debbie A. D. C. Jaarsma, Erik Heineman, Renée A. Scheepers

**Affiliations:** 1 Department of Surgery, University of Groningen, The Netherlands; 2 Department of Surgery, Amsterdam University Medical Center, The Netherlands; 3 Professorship of Sustainable Finance and Entrepreneurship of the Hanzehogeschool Groningen, The Netherlands; 4 Section Socio-Medical Sciences of the Erasmus School of Health Policy & Management, Rotterdam, The Netherlands; 5 Department of Medical Psychology, Amsterdam University Medical Centers, University of Amsterdam, The Netherlands; 6 Amsterdam Public Health Research Institute, Amsterdam, The Netherlands; 7 Utrecht University, Utrecht, The Netherlands; 8 Department of Surgery, University Medical Center Groningen, The Netherlands

## Abstract

**Background:**

Workplace learning of health care professionals benefits from a cyclical process of self-regulated learning (SRL), in the phases of forethought, performance, and reflection. This SRL process can reduce safety incidents, a particular concern in high-risk situations of surgical practice. Surgeons who endorse a growth mindset and are motivated professionals may engage more actively in SRL. However, the interrelations between mindset, motivation, and SRL remain unclear. Therefore, we investigated how surgeons’ mindset is associated with SRL, and whether this association is mediated by motivation.

**Methods:**

We invited surgeons of Dutch surgical associations to complete a web-based survey containing validated instruments on growth and fixed mindset, autonomous and controlled motivation, and the three phases of SRL. Data were analyzed using path analysis in a sample of 170 surgeons.

**Results:**

Growth mindset was positively associated with all three phases of SRL: forethought (β = 0.30, 95% CI [0.164, 0.441]), performance (β = 0.22, 95% CI [0.076, 0.373]), and reflection (β = 0.18, 95% CI [0.040, 0.323]). Additionally, fixed mindset was indirectly negatively associated with the forethought phase of SRL through lower autonomous motivation (β = –0.03, BC 95% CI [–0.082, –0.002]).

**Discussion:**

Surgeons holding a stronger fixed mindset reported lower levels of autonomous motivation, which were subsequently associated with less frequent use of SRL in the forethought phase. Conversely, more frequent use of SRL across its three phases was reported by surgeons holding a stronger growth mindset. These findings call for support of surgeons’ growth mindset, to facilitate surgeons’ roles as motivated and self-regulating learners striving for continuous performance improvement.

## Introduction

Lifelong learning is recognized as a professional responsibility of health care professionals to support their professional stewardship, maintain competence, and foster both their personal and professional growth [1]. This process of lifelong learning supports health care professionals’ involvement in continuous quality improvement and stimulates them in proactively recognizing and preventing risks to patient safety [2,3]. These risks are particularly challenging to prevent in high-risk surgical situations, as surgical-related safety incidents are common causes of adverse events in hospitals [4,5]. In this context, it has been widely acknowledged that surgical-related safety incidents should be prevented by improving safety culture, education, and adhering to quality improvement and learning cycles [6,7]. How surgeons’ own learning cycles may benefit from their mindset and motivation is, however, poorly understood. Understanding how their mindset and motivation stimulates surgeons to learn in their daily practice is important for fostering this culture of continuous learning and, ultimately, improving patient care. To contribute to this body of knowledge, this study explores the associations between mindset, motivation, and surgeons’ learning behaviors.

Learning behaviors in daily practice are widely studied by the concept of self-regulated learning (SRL) [8]. This concept describes a cyclical view of learning in which professionals self-regulate their learning in three phases: forethought, performance, and reflection [9]. The forethought phase involves goal setting, planning, and strategizing before starting a learning task. The performance phase includes actively engaging with the material, using chosen strategies, and monitoring progress, while this performance is evaluated in the reflection phase by seeking feedback and planning for future learning based on insight gained [8,9]. An SRL-based surgical workforce that continuously reflects and learns from what goes well and what needs improvement likely contributes to surgeons’ engagement in proactive behaviors and bottom-up quality and safety improvements [2,7,10,11].

SRL is known to be facilitated by a learning mindset focusing on growth [12,13,14,15]. A growth mindset is characterized by the belief that abilities and intelligence can be developed through effort and persistence, as opposed to a fixed mindset that assumes these qualities are innate and unchangeable [12]. Growth mindset-oriented individuals embrace effort, are resilient in experiencing setbacks, reflect on their actions, seek feedback, learn from criticism, and find inspiration in others’ success [12,13,14,15]. Fixed mindset-oriented individuals, in contrast, tend to focus on proving their abilities to others to demonstrate their competence, while simultaneously avoiding challenges, ignoring criticism, and interpreting the success of others as threatening [15,16,17]. In the context of learning from adverse events and medical errors, neuropsychological research shows that those with a growth mindset have increased brain activity when something goes wrong and learn more, while fixed mindset individuals learn less or nothing from the event [18,19]. The potential application of mindset theory to surgical education has been described [20,21]; however, empirical evidence of mindset and its association with SRL in the context of surgical education is limited [22,23].

Research has indicated that a growth mindset can enhance both learning and motivation in educational settings [24,25]. Motivation, as described by the Self-Determination theory, ranges from amotivation to introjected motivation, progressing to extrinsic motivation, and culminating in identified and intrinsic motivation [26]. Introjected and extrinsic forms of motivation are considered as controlled motivation, while autonomous motivation combines identified and intrinsic motivation [26]. Autonomous motivation entails undertaking an activity because of the pleasure associated with it; the values underlying the behavior are endorsed, or these values are integrated in one’s sense of self [26,27,28]. Autonomously motivated surgeons tend to show proactive behaviors and initiate quality improvement that contribute to SRL in daily practice [29]. Moreover, when those with a growth mindset perceive challenges as growth opportunities, their autonomous motivation might reinforce their commitment to SRL as it stems from the belief in self-improvement [27,29,30]. Autonomous motivation might therefore mediate the association between growth mindset and learning in surgeons.

Controlled motivation, in contrast, involves engaging in an activity due to its instrumental value; a person experiences external pressure to think, feel, or act in a specific way [26]. Within and beyond the healthcare setting, controlled motivation has been described to be effective for specific core task behaviors, which are often relatively simple, mundane, and repetitive, such as following a hygiene protocol [29,31]. In the long term, however, behaviors driven by controlled motivation might vanish once the desirability of the reward disappears [26]. SRL often requires persistent effort, while direct rewards for this behavior may be absent. SRL may therefore not necessarily benefit from controlled motivation, while it is unclear whether controlled motivation might mediate the association between mindset and learning.

**Figure 1:**
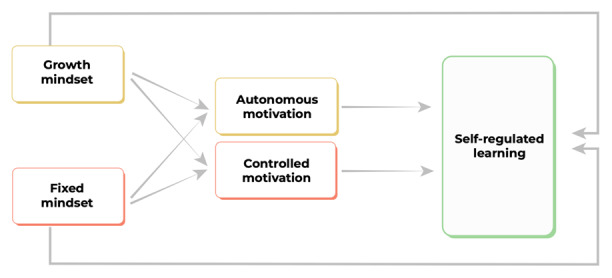
Conceptual model on the associations between mindset, motivation, and self-regulated learning.

Thus, while prior studies have discussed the potential mechanisms of mindset in surgical education, the exact associations between mindset, motivation, and learning behaviors in surgeons remain understudied. Specifically, it is unclear whether and how the association of growth and fixed mindset with SRL may be due to autonomous and controlled motivation. To address this knowledge gap, we investigated in this study the association between mindset and SRL in surgeons and explored the potential mediating role of autonomous versus controlled motivation herein (see Figure 1). Our findings may offer new insights and strategies in fostering effective learning in surgeons, contributing to improved quality and safety of patient care.

## Methods

### Study population and setting

We invited surgeons from the national associations of surgical subspecialties in General Surgery, Orthopaedic Surgery, Gynaecology and Obstetrics, Plastic Surgery, Urology, ENT, and Thoracic Surgery in the Netherlands to participate in the study and complete a web-based survey (see Survey design). The survey was offered via a web-based platform, where each participant could create a personal account and complete the survey.

The invitation to complete the survey was communicated in newsletters and on the website of the different surgical associations to all their members (n = 3,500). The invitation included an explanation of the research project and a web link to generate a personal account on the website to complete the survey. As the survey was distributed on a national level, demographic variety and the inclusion of academic, teaching, and non-teaching hospitals were facilitated. After creating an account on the website, the purpose of the study was explained once more, and the anonymous participants were asked to give their consent electronically before being offered the survey questions. Data collection took place from January 2021 until November 2022.

### Survey design

We designed the survey based on a literature search and focus group session. In the literature search, we collected existing instruments with demonstrated validity evidence on the measurement of mindset, motivation, and SRL. The quality of the papers publishing those instruments was assessed using the Standard Quality Assessment Criteria for Evaluating Primary Research Papers, and those with the highest score per concept were selected [32]. The selected surveys were then translated independently into Dutch by two researchers (KD and GV) through forward-backward translation. Their clarity and relevance to surgical practice were evaluated in a focus group session with nine surgeons. Participants’ experiences and reflections on the survey were discussed, as well as the clarity and relevance of the items to surgical practice. In this context, participants considered most original measurements suitable and clear to assess mindset, motivation, and SRL, yet only the SRL performance phase required rephrasing of items to surgical practice. For this scale, suggestions for rephrasing the item were discussed with the participants and the research team, who decided on the final rephrasing or removal of an item (see Measurements). The resulting survey (see Supplementary Table 1) was then presented on a web-based platform, where members of the research team checked survey flow before distribution.

### Measurements

Mindset was assessed using the questionnaire designed by El-Fattah et al. to measure growth (incremental) and fixed (entity) mindsets with seven items each [33]. The items were completed on a 4-point Likert-type scale ranging from strongly disagree (1) to strongly agree (4). The respective items were averaged, with higher scores indicating a more growth and fixed mindset, respectively.

Motivation was measured by the Multidimensional Work Motivation Scale (MWMS) developed by Gagné et al., which was previously validated in an international sample of professionals for the measurement of autonomous and controlled work motivation [34]. Autonomous motivation was measured as the combination of intrinsic (three items) and identified (three items) motivation, whereas controlled motivation was measured as the combination of extrinsic (six items) and introjected (four items) motivation. Items were answered on a 7-point Likert-type scale ranging from not at all (1) to completely (7). The respective items were averaged, with higher scores indicating more autonomous and controlled motivation, respectively.

Self-regulated learning (SRL) was measured by the self-regulated workplace learning instrument developed by Fontana et al., adjusted from the original SRL questionnaire designed by Zimmerman et al. to be able to assess SRL in a specific workplace setting [8,9]. This scale was used to measure the forethought phase with seven items and the reflection phase with three items. The performance phase of SRL was assessed using the Workplace Learning Activity (WLA) scale [8]. This scale consisted of 11 items, which were adjusted to the surgical context (see Survey design). Specifically, following the input of the focus group session, three items that were not applicable to surgical practice were deleted (e.g., “Finding a better way to do a task by trial and error”), and eight items were rephrased to better fit the context of surgical practice; for example, “Asking colleagues for advice” was rephrased into “Asking a colleague to give feedback on my technical skills” (see Supplementary Table 1). Overall, the three SRL phases were assessed by 18 items, which asked respondents to indicate the frequency of their learning activities during the past three months on a 5-point Likert-type scale ranging from almost never (1) to nearly always (5). The respective items were averaged, with higher scores indicating more frequent SRL behavior.

Lastly, the survey inquired on demographic information about gender, years of experience since medical school graduation, hospital type (academic hospital, general teaching hospital, general non-teaching hospital, or private clinic), type of contract (hospital-employed, self-employed, combination of hospital- and self-employed, or differently employed), and subspecialty.

### Data Analysis

Data were analyzed using SPSS 28 and Mplus 8.9. Reliability of all measurement scales was assessed according to internal consistency (satisfactory when Cronbach’s alpha ≥ 0.70) [35]. Sample characteristics were represented using frequencies and descriptive statistics. Bivariate associations between the study variables were examined by Pearson correlation analysis. Gender and years of experience were included in the correlation analysis to assess whether they were potential confounders and included in the subsequent path analysis in case of a correlation with one of the study variables.

The proposed mediation model was tested by path analysis using maximum likelihood estimation. The model included growth and fixed mindset as independent variables, autonomous and controlled motivation as parallel mediators, and the forethought, performance, and reflection phases of SRL as dependent variables. Standardized path coefficients (β) were reported along with their p-values and confidence intervals. The significance of the indirect effects of the independent variables on the dependent variables via the mediators was estimated by bootstrapping with 10, 000 bootstrap samples, as recommended by Hayes [36]. Accordingly, standardized coefficients (β) are reported together with their bias-corrected bootstrap confidence intervals.

**Figure 2:**
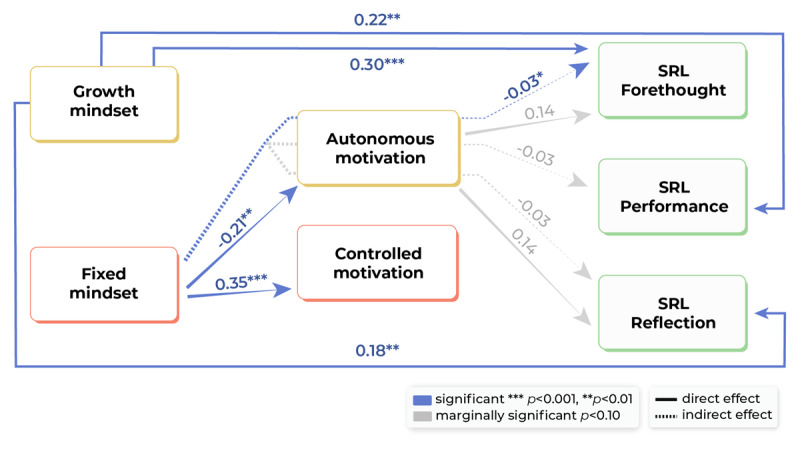
Results of the mediation path analysis. Only (marginal) significant paths are displayed. Standardized path coefficients are reported for direct and indirect effects (represented by separate arrows). Total effects are not reported in Figure 2 but in Supplementary Table 3. The control path of years of work experience was omitted for figure clarity.

We reported associations when they were statistically significant at the 5% level, as indicated by 95% confidence intervals that do not include zero. In addition, we report marginally significant associations at the 10% level, as indicated by 90% confidence intervals that exclude zero, to provide a comprehensive and nuanced overview of both statistically robust and potentially meaningful associations. This approach is particularly relevant in exploratory studies with relatively small sample sizes and limited statistical power for testing complex models [37,38,39]. This was the case in our study, which included a sample size of N = 170 and a path model with two independent variables, two mediators, and three dependent variables, and one control variable (see Figures 1 and 2).

Due to the cross-sectional data, the mediation analysis cannot reveal any evidence of causality but solely reveal atemporal associations [40].

### Ethics Statement

The study was conducted in line with the principles of the Declaration of Helsinki [41], and the institutional ethical review board of the Academic Medical Centre of the University of Groningen (UMCG) waived ethical approval for this study (#2018 00834). All input gathered during the focus group sessions, as well as the transcripts of the focus group sessions, were anonymized. Written informed consent was obtained from all participants before the start of the focus group. Before completing the survey, the voluntary and anonymous nature of the study was explained, and respondents were asked to sign their consent electronically.

## Results

### Sample description

In total, 213 surgeons responded to the survey, after which 43 were excluded due to missing data on outcomes (n = 23), mediators (n = 13), or type of contract (n = 6); input on the latter variable was necessary to determine whether respondents were practicing (rather than retired) surgeons. The remaining 170 surgeons were included for analysis, of which 89 (52.4%) identified as female, 80 (47.1%) as male, and one as non-binary (0.6%). They reported an average of 22.43 years (SD = 11.66) of work experience. The sample consisted of 63 (37.1%) gynecologists, 23 (13.5%) general surgeons, 40 (23.5%) orthopedic surgeons, 10 (5.9%) urologists, 3 (1.8%) plastic surgeons, 5 (2.9%) thoracic surgeons, and 2 (1.2%) ENT surgeons. See Supplementary Table 2 for further sample characteristics.

### Reliability of measures and bivariate associations between study variables

The levels of internal consistency for most measures on mindset, motivation, and SRL were satisfactory to good (Cronbach’s alphas ranging from 0.74 to 0.83; see Table 1). The internal consistency of fixed mindset and SRL reflection was slightly beyond the satisfactory level of 0.70 (Cronbach’s alphas of 0.67; see Table 1; see Discussion).

**Table 1:** Descriptive Statistics of and Bivariate Associations between Study and Potential Control Variables (N = 170).


STUDY VARIABLES	1	2	3	4	5	6	7	*M (SD)*	CRONBACH’S ALPHA

1 Growth mindset^a^		–0.09	0.13	0.07	0.33***	0.20*	0.24**	2.95 (0.42)	0.80

2 Fixed mindset^a^			–0.22**	0.35***	0.01	–0.01	–.00	2.14 (0.39)	0.67

3 Autonomous motivation^b^				–0.05	0.19*	0.15(*)	0.15(*)	5.89 (0.72)	0.74

4 Controlled motivation^b^					0.04	0.03	0.03	3.48 (1.00)	0.83

5 SRL Forethought^c^						0.36***	0.56***	3.43 (0.62)	0.78

6 SRL Performance^c^							0.35***	3.23 (0.59)	0.77

7 SRL Reflection^c^								3.50 (0.69)	0.67

**Control variables**									

8 Work experience in years	0.04	–0.04	0.10	–.14(*)	0.28***	–0.00	0.14(*)	22.43 (11.66)	–

9 Gender^d^	0.08	0.02	–0.05	–0.08	0.00	–0.05	0.03	–	–


Note. ^a^scale range 1–4; ^b^scale range 1–7; ^c^scale range 1–5; ^d^dichotomous 1 = female, 2 = male. Coefficients between continuous variables 1–8 represent Pearson correlation coefficients. Coefficients involving the dichotomous variable 9 represent point-biserial correlation coefficients. ^(*)^*p* < 0.10, *p < 0.05, **p < 0.01, ***p < 0.001.

The correlation analysis (see Table 1) revealed that growth mindset showed no associations with autonomous or controlled motivation, while fixed mindset was significantly negatively associated with autonomous motivation (r = –0.22, p = 0.003) and significantly positively associated with controlled motivation (r = 0.35, p < 0.001). Additionally, growth mindset showed significant positive associations with all SRL phases, i.e., forethought (r = 0.33, p < 0.001), performance (r = 0.20, p = 0.011), and reflection (r = 0.24, p = 0.001), while fixed mindset did not show associations with the SRL phases. Autonomous motivation was significantly positively associated with SRL in the forethought phase (r = 0.19, p = 0.014), and marginally significantly positively associated with the performance and reflection phases of SRL (r = 0.15, p = 0.050 and r = 0.15, p = 0.051, respectively). Controlled motivation was not associated with the three SRL phases.

### Associations between mindset, motivation and SRL

The results of the path analysis on associations between mindset, motivation and SRL, controlling for years of experience, are displayed in Figure 2 and Supplementary Table 3.

Growth mindset was significantly positively directly associated with all three phases of SRL, forethought (β = 0.30, 95% CI [0.164, 0.441]), performance (β = 0.22, 95% CI [0.076, 0.373]), and reflection (β = 0.18, 95% CI [0.040, 0.323]). This indicates that surgeons with a stronger growth mindset reported more frequent use of SRL. In contrast, no significant direct effects of fixed mindset on SRL phases were found.

Furthermore, growth mindset was not associated with autonomous or controlled motivation, whereas fixed mindset was significantly negatively associated with autonomous motivation (β = –0.21, 95% CI [–0.362, –0.061]) and significantly positively associated with controlled motivation (β = 0.35, 95% CI [0.219, 0.485]). This indicates that surgeons with a stronger fixed mindset reported lower levels of autonomous motivation and higher levels of controlled motivation.

Autonomous motivation was marginally significantly positively associated with the forethought and reflection phases of SRL (β = 0.14, 90% CI [0.018, 0.261]; β = 0.14, 90% CI [0.005, 0.270], respectively), whereas controlled motivation was not associated with the three SRL phases. This indicates that surgeons with higher levels of autonomous motivation tended to report more frequent use of planning and reflection strategies.

No significant indirect effects of growth mindset via autonomous or controlled motivation on the three SRL phases were found. However, a significant negative indirect effect of fixed mindset on the forethought phase of SRL via autonomous motivation was found (β = –0.03, 95% CI [–0.082, –0.002]). This indicated that surgeons with a stronger fixed mindset reported lower levels of autonomous motivation, which were subsequently associated with less frequent use of SRL planning strategies. For SRL performance and reflection strategies, similar negative indirect effects were observed, although these were only marginally significant (β = –0.03, 90% CI [–0.069, –0.002]; β = –0.03, 90% CI [–0.077, –0.003], respectively).

## Discussion

### Main findings

This study showed that surgeons with a stronger growth mindset more frequently reported using SRL across the forethought, performance, and reflection phases. In contrast, surgeons with a stronger fixed mindset reported lower levels of autonomous motivation, which were subsequently associated with less frequent use of SRL in the forethought phase.

### Explanation of findings

In the interpretation of our findings, it is crucial to underscore that mindset does not adhere to a simple dichotomy [12]. In the educational setting, research has shown that mindsets are context-dependent, allowing individuals to exhibit varying mindsets based on the type of learning tasks they encounter [12,42]. This is supported by our finding that there is no inherent negative association between a fixed and a growth mindset. Therefore, the presence of a pronounced fixed mindset in an individual does not necessarily exclude the presence of a growth mindset and vice versa.

Growth mindset was directly associated with more frequent use of SRL, aligning with previous findings showing that growth mindset-oriented learners are more perseverant in their learning process, more active in seeking feedback, and more receptive to feedback—all of which are crucial elements of SRL [14]. On the other hand, SRL was not directly affected by fixed mindset. However, fixed mindset was indirectly associated with less frequent use of SRL through lower levels of autonomous motivation. Autonomous motivation, based on the Self-Determination theory, is characterized by a pleasurable connection with one’s work while professional values are integrated in one’s professional identity [24,26,28]. Professionals are less likely to perceive such connection and integration in their work—demonstrated by limited autonomous motivation—when holding a stronger fixed mindset. Fixed mindset-oriented professionals are more inclined to pursue performance goals while aiming to gain positive assessments and preventing negative ones, thereby focusing on external judgements rather than on internalizing professional values—a process necessary for autonomous motivation [14,42]. Supporting autonomous motivation among surgeons holding a stronger fixed mindset should be considered, especially since more autonomous motivation among fixed mindset-oriented surgeons may facilitate their use of SRL strategies [42,43].

SRL was not affected by controlled motivation in a negative nor positive way. Prior research in the educational setting has shown that individuals with high levels of controlled motivation are focused on receiving external rewards and avoiding negative feelings such as shame, and are less likely to involve in deep-learning strategies [24,43]. These, as well as our findings, might be explained by controlled motivation encouraging performance on core tasks and behavior in line with certain laws and protocols, rather than “going the extra mile,” such as exhibiting proactive (learning) behaviors at work [24,25,26,27]. These proactive behaviors, such as voicing behaviors, i.e., speaking up or acting in the face of threats to patient safety, and actively participating or initiating quality improvements are stimulated by autonomous motivation [10,29,31]. To exercise lifelong learning and to stimulate continuous quality improvement, surgeons with high levels of controlled motivation might benefit from developing a growth mindset and a work environment that stimulates their autonomous motivation [44,45].

A study in the field of neurosurgery revealed that both residents and neurosurgeons exhibit a fixed mindset regarding intelligence, in contrast to medical students or the general population [23]. As a mindset is known to be context-dependent, this inclination could potentially be attributed to medical students adopting a fixed perspective as they advance in their surgical careers. One of the challenges herein might arise when there is a mismatch between the mindset of the learner and the educator [46,47]. For instance, a trainee with a growth mindset receiving feedback from an educator with a fixed mindset, or being surrounded by educators who do not openly acknowledge their ongoing learning journeys, might impact the mindset of the trainee [16]. In addition, the surgical culture that was long known for its strong beliefs and traditions and as a profession where errors were less tolerable due to their significant impact on patient outcomes might indirectly favor a fixed-mindset-oriented surgical workforce. However, recent developments in creating a just culture, including the vision to collectively learn from adverse events, may help to promote a growth mindset in surgeons [10,48].

### Strengths and limitations

To the best of our knowledge, this is the first empirical study investigating how the relationship between mindset and SRL in the surgical setting is mediated by motivation. We addressed this knowledge gap based on a national study using validated instruments which were tailored to the surgical setting based on a focus group. A few limitations must be considered when interpreting the findings of this study. First, given the small sample size and the fact that the study sample only contained surgeons working in the Dutch healthcare system, the generalizability of the results is limited. It is unclear how many surgeons actually read the newsletters that were used to distribute the survey, and participation was voluntary. Ultimately, 5% of the study population participated, and it cannot be excluded that scale scores on mindset, motivation, or SRL may have been affected by selection bias. Nonetheless, this might not have necessarily influenced the direction or magnitude of the relationships between the concepts under study; these were consistent with empirical and theoretical support for associations between mindset, motivation, and learning in other (non-medical) settings [12,13,14,15,24,25]. In addition, no causal associations could be assessed due to the cross-sectional nature of this study [40].

The survey was performed based on validated questionnaires and most scales used showed satisfactory to good internal consistency. However, the internal consistency of two subscales (fixed mindset and SRL reflection) fell slightly below the satisfactory threshold. Therefore, the findings should be interpreted with this limitation in mind, although only a minimal impact on the results is expected given the near-satisfactory internal consistency of the respective subscales. Furthermore, the combination of scales in the survey enabled our study to innovate insights into associations between mindset, motivation, and self-regulated workplace learning, which had not yet been studied in a (surgical) faculty population in the context of continuing medical education. The marginally significant effects that were found in addition to the significant ones, despite the small sample size [38], indicate the relevance for future studies investigating these associations at a larger scale. Such efforts would also benefit more firm conclusions about the mediating role of motivation between growth mindset and SRL—a mediating association that we did not find, but a larger study could clarify whether this may be due to limited power.

### Implications and future directions

Medical practice, and surgical practice specifically, represents a unique learning environment, in which the ideal learner is one who understands the learning context, utilizes available resources and opportunities for growth, and constantly reflects on his or her own performance as well as the group performance of everyone involved in providing surgical care [45]. SRL is therefore a critical skill for surgical health care professionals and involves the ability to set goals, plan strategies, monitor progress, and make necessary adjustments to achieve desired outcomes [8,45]. This study shows that a growth mindset is associated with better SRL in surgeons. Considering that researchers have previously reported that a person’s mindset is malleable [42,49], this opens up the avenue to stimulate quality improvements through surgeons’ growth mindset.

Future research could focus on tailoring growth mindset interventions used in the educational context towards the surgical setting [49]. Providing targeted feedback that rewards effort, focuses on strategy use and progress rather than solely on outcomes has proven effective in shifting individuals’ perspectives towards a growth-oriented mindset [22,23,49]. In addition, incorporating growth mindset principles into educational curricula, by highlighting the brain’s adaptability and capacity for learning, can encourage (future) surgeons to see their abilities as malleable and subject to improvement [21,49]. Simultaneously, it is necessary to reevaluate current initiatives that seek to motivate surgeons extrinsically to improve specific quality outcomes by incentivizing or penalizing. It is recommended that these incentive-oriented approaches are enhanced by integrating elements that also encourage autonomous motivation. This entails factors like aligning with the inherent motivations and values of surgeons, granting autonomy, providing supportive supervision and feedback, demonstrating appreciation and respect, fostering positive interpersonal connections, and offering opportunities for personal and professional growth [2,29].

Future research could differentiate core task behaviors from proactive behaviors in the surgical learning context to better understand the difference between autonomously versus controlled motivated surgeons and their intent to actively participate in quality and safety improvement initiatives, and further link these concepts to patient outcomes. Finally, it is worth noting that while a growth mindset and autonomous motivation can be advantageous, it does not guarantee success nor eliminate the challenges faced by surgeons. Surgery requires a combination of technical skills, knowledge, experience, and a commitment to ongoing improvement. However, cultivating a growth mindset can contribute to a surgeon’s professional development and SRL. Aside of SRL, other processes may clarify mindset and motivation, and therefore, future research should also uncover other possible determinants of SRL—for example, learner readiness—building on state-of-the-art insights into SRL in medical education [50,51].

## Conclusions

This study advances previous research by providing novel insights into how motivation mediates the association between mindset and SRL in surgical practice. Specifically, surgeons holding a fixed mindset were less autonomously motivated professionals who reported less frequent use of SRL, while more frequent use of SRL was reported by surgeons holding a stronger growth mindset. SRL can specifically benefit from a growth mindset promoting a willingness to embrace challenges, learn from failures, and seek out new knowledge and techniques. This empowers surgeons to take ownership of their learning, engage in self-directed activities, and contribute to a culture of shared knowledge. Therefore, promoting a growth mindset may be considered in order to facilitate the process of self-regulated learning, which is crucial for surgeons’ adaptability and innovation—ultimately leading to improved patient outcomes.

## Additional File

The additional file for this article can be found as follows:

10.5334/pme.2144.s1 Supplementary Tables. Supplementary Tables 1–3.
