# Supplementary material for: Exploring Workplace Learning in Surgical Practice: How Mindset and Motivation Are Associated With Self-Regulated Learning Behaviors
Source: Perspect Med Educ. 2025 Nov 3;14(1):699–709. doi: 10.5334/pme.2144 (PMC12594085; doi:10.5334/pme.2144)
Supplement: Supplementary Tables. — Supplementary Tables 1–3. [file pme-14-1-2144-s1.pdf]

Dabekaussen et al. (2025). Exploring workplace learning in surgical practice: How mindset and motivation are associated with self-regulated learning behaviors. *Perspectives on Medical Education*. DOI: https://doi. org/10.5334/pme.2144

Supplementary table 1. The mindset, motivation, and self-regulated learning items

Main construct	Item
Mindset	You are born with a fixed amount of intelligence.
	Good performance in a task is a way of showing others that you are intelligent.
	You have a certain amount of intelligence and you cannot do much to change it.
	If you fail in a task, you question your intelligence.
	When you exert a lot of effort, you show that you are not intelligent.
	Difficulties and challenges prevent you from developing your intelligence
	Your abilities are determined by how intelligent you are.
	Good preparation before performing a task is a way to develop your intelligence.
	Performing a task successfully can help develop your intelligence.
	You can develop your intelligence if you really try
	When you learn new things, your basic intelligence improves.
	The effort you exert, improves your intelligence.
	If you fail a task, you still trust your intelligence.
	Criticism from others can help develop your intelligence.
Motivation	I put efforts into my current job to get others' approval (e.g. supervisor, colleague, family, patients)
	I put efforts into my current job because others will respect me more (e.g. supervisor, colleague, family, patients)
	I put efforts into my current job to avoid being criticized by others (e.g. supervisor, colleague, family, patients)
	I put efforts into my current job because others will reward me financially only if I put enough effort in it (e.g. employer, supervisor)
	I put efforts into my current job because others offer me greater job security if I put enough effort in it (e.g. employer, supervisor)
	I put efforts into my current job because I risk losing my job if I don't put enough effort in it
	I put efforts into my current job because I have to prove to myself that I can
	I put efforts into my current job because it makes me feel proud of myself
	I put efforts into my current job because otherwise I will feel ashamed of myself
	I put efforts into my current job because I will feel bad about myself

	I put efforts into my current job because I personally consider it important
	I put efforts into my current job because it aligns with my personal values
	I put efforts into my current job because it has personal significance to me
	I put efforts into my current job because I have fun doing my job
	I put efforts into my current job because what I do in my work is exciting
	I put efforts into my current job because what I do in my work is interesting
Self-regulated learning (SLR)	I use specific strategies for different type of things I need to learn
	I ask myself questions about each learning task before I begin
	When planning my learning, I adapt strategies that have worked in the past
	I set goals to help me manage the time I spend learning
	I set realistic deadlines for learning when I have identified a learning need
	I set long-term goals (monthly or yearly) for myself in order to direct my learning activities
	I think of several ways to solve a problem and choose the best one
	I ask myself if there were other ways to do things after I finished the task
	I think about what I have learned after I finish
	I know how well I have learned once I have finished a task
	Asking help from a colleague to learn from it
	Discussing incidents to learn from them
	Asking a colleague to give feedback on my technical skills
	Asking a colleague to give feedback on my non-technical skills
	Giving a colleague feedback on their technical skills
	Giving a colleague feedback on their non-technical skills
	Attend a surgery of a colleague
	Asking patients for feedback on my technical or non-technical skills

Dabekaussen et al. (2025). Exploring workplace learning in surgical practice: How mindset and motivation are associated with self-regulated learning behaviors. *Perspectives on Medical Education*. DOI: https://doi. org/10.5334/pme.2144

Supplementary table 2. Demographics of study sample (*N* = 170)

**Demographic**		**Frequency (%)***
**Gender**	Male	80 (47.1%)
	Female	89 (52.4%)
	Non-binary	1 (0.6%)
**Hospital type**	Academic hospital	35 (20.6%)
	General teaching hospital	103 (60.6%)
	General non-teaching hospital	21 (12.4%)
	Private clinic	11 (6.5%)
**Type of contract**	Hospital employed	87 (51.2%)
	Self-employed (partnership)	66 (38.8%)
	Combination hospital/self-employed	4 (2.4%)
	Other type of employment	13 (7.6%)
**Specialty**	General surgery	23 (13.5%)
	Gynaecology & Obstetrics	63 (37.1%)
	Orthopaedic surgery	40 (23.5%)
	Urology	10 (5.9%)
	Plastic Surgery	3 (1.8%)
	Thoracic Surgery	5 (2.9%)
	ENT Surgery	2 (1.2%)

Dabekaussen et al. (2025). Exploring workplace learning in surgical practice: How mindset and motivation are associated with self-regulated learning behaviors. *Perspectives on Medical Education*. DOI: https://doi. org/10.5334/pme.2144

Supplementary table 3. Standardized path coefficients (β), p-values, 95% and 90% confidence intervals (CI) following from the mediation path analysis for total, direct, and indirect effects of the independent variables (growth mindset and fixed mindset) on the dependent variables (SRL forethought, performance and reflection) via mediators (autonomous motivation and controlled motivation)

Effect type	Independent variable → mediator / outcome variable	β	p[a]	95% CI[b]	90% CI[c]
Total	Growth mindset → SRL forethought	0.32	<0.001	**[0.193, 0.449]**	[0.214, 0.428]
Total	Growth mindset → SRL performance	0.24	0.001	**[0.097, 0.382]**	[0.120, 0.359]
Total	Growth mindset → SRL reflection	0.20	0.004	**[0.062, 0.331]**	[0.084, 0.310]
Total	Fixed mindset → SRL forethought	0.05	0.546	[-0.109, 0.205]	[-0.083, 0.180]
Total	Fixed mindset → SRL performance	0.02	0.745	[-0.122, 0.170]	[-0.098, 0.147]
Total	Fixed mindset → SRL reflection	0.01	0.924	[-0.145, 0.16,]	[-0.121, 0.135]
Direct	Growth mindset → SRL forethought	0.30	<0.001	**[0.164, 0.441]**	[0.187, 0.418]
Direct	Growth mindset → SRL performance	0.22	0.003	**[0.076, 0.373]**	[0.100, 0.349]
Direct	Growth mindset → SRL reflection	0.18	0.012	**[0.040, 0.323]**	[0.063, 0.300]
Direct	Fixed mindset → SRL forethought	0.06	0.480	[-0.113, 0.241]	[-0.085, 0.213]
Direct	Fixed mindset → SRL performance	0.04	0.643	[-0.129, 0.209]	[-0.102, 0.182]
Direct	Fixed mindset → SRL reflection	0.03	0.693	[-0.133, 0.200]	[-0.106, 0.173]
Direct	Growth mindset → autonomous motivation	0.10	0.144	[-0.035, 0.242]	[-0.013, 0.220]
Direct	Growth mindset → controlled motivation	0.11	0.142	[-0.035, 0.247]	[-0.013, 0.224]
Direct	Fixed mindset → autonomous motivation	-0.21	0.006	**[-0.362, -0.061]**	[-0.338, -0.085]
Direct	Fixed mindset → controlled motivation	0.35	0.000	**[0.219, 0.485]**	[0.240, 0.464]

Direct	Autonomous motivation → SRL forethought	0.14	0.059	[-0.005, 0.284]	**[0.018, 0.261]**
Direct	Autonomous motivation → SRL performance	0.12	0.127	[-0.034, 0.271]	[-0.009, 0.247]
Direct	Autonomous motivation → SRL reflection	0.14	0.088	[-0.020, 0.296]	**[0.005, 0.270]**
Direct	Controlled motivation → SRL forethought	0.04	0.575	[-0.099, 0.178]	[-0.076, 0.155]
Direct	Controlled motivation → SRL performance	0.03	0.723	[-0.121, 0.174]	[-0.097, 0.151]
Direct	Controlled motivation → SRL reflection	0.01	0.920	[-0.155, 0.172]	[-0.129, 0.145]
Indirect	Growth mindset → SRL forethought (via autonomous motivation)	0.01	-	[-0.004, 0.053]	[-0.001, 0.045]
Indirect	Growth mindset → SRL performance (via autonomous motivation)	0.01	-	[-0.004, 0.052]	[-0.001, 0.045]
Indirect	Growth mindset → SRL reflection (via autonomous motivation)	0.01	-	[-0.004, 0.055]	[-0.001, 0.047]
Indirect	Growth mindset → SRL forethought (via controlled motivation)	0.00	-	[-0.009, 0.032]	[-0.006, 0.027]
Indirect	Growth mindset → SRL performance (via controlled motivation)	0.00	-	[-0.012, 0.030]	[-0.008, 0.025]
Indirect	Growth mindset → SRL reflection (via controlled motivation)	0.00	-	[-0.018, 0.029]	[-0.013, 0.023]
Indirect	Fixed mindset → SRL forethought (via autonomous motivation)	-0.03	-	**[-0.082, -0.002]**	[-0.073, -0.006]
Indirect	Fixed Mindset → SRL performance (via autonomous motivation)	-0.03	-	[-0.079, 0.002]	**[-0.069, -0.002]**
Indirect	Fixed Mindset → SRL reflection (via autonomous motivation)	-0.03	-	[-0.087, 0.000]	**[-0.077, -0.003]**
Indirect	Fixed Mindset → SRL forethought (via controlled motivation)	0.01	-	[-0.037, 0.065]	[-0.028, 0.055]
Indirect	Fixed Mindset → SRL performance (via controlled motivation)	0.01	-	[-0.044, 0.065]	[-0.034, 0.054]
Indirect	Fixed Mindset → SRL reflection (via controlled motivation)	0.00	-	[-0.055, 0.062]	[-0.045, 0.053]

Note. The analysis was adjusted for years of experience. Confidence intervals in bold text represent the (marginally) significant ones.

[a] *p*-values are not reported for indirect effects, due to bootstrapping of 10.000 bootstrap samples.

[b,c] confidence intervals of indirect effects represent bias-corrected bootstrap confidence intervals.
